# WEBnm@: a web application for normal mode analyses of proteins

**DOI:** 10.1186/1471-2105-6-52

**Published:** 2005-03-11

**Authors:** Siv Midtun Hollup, Gisle Salensminde, Nathalie Reuter

**Affiliations:** 1 Computational Biology Unit, Bergen Center for Computational Science, University of Bergen, Thormøhlensgt.55, N-5008 Bergen, Norway

## Abstract

**Background:**

Normal mode analysis (NMA) has become the method of choice to investigate the slowest motions in macromolecular systems. NMA is especially useful for large biomolecular assemblies, such as transmembrane channels or virus capsids. NMA relies on the hypothesis that the vibrational normal modes having the lowest frequencies (also named soft modes) describe the largest movements in a protein and are the ones that are functionally relevant.

**Results:**

We developed a web-based server to perform normal modes calculations and different types of analyses. Starting from a structure file provided by the user in the PDB format, the server calculates the normal modes and subsequently offers the user a series of automated calculations; normalized squared atomic displacements, vector field representation and animation of the first six vibrational modes. Each analysis is performed independently from the others and results can be visualized using only a web browser. No additional plug-in or software is required. For users who would like to analyze the results with their favorite software, raw results can also be downloaded. The application is available on . We present here the underlying theory, the application architecture and an illustration of its features using a large transmembrane protein as an example.

**Conclusion:**

We built an efficient and modular web application for normal mode analysis of proteins. Non specialists can easily and rapidly evaluate the degree of flexibility of multi-domain protein assemblies and characterize the large amplitude movements of their domains.

## Background

Molecular modeling provides several powerful tools for computing the dynamics of proteins. Normal Mode Analysis (NMA) is a well suited approach to study dynamics of proteins, especially when the protein is relatively big (several thousand amino acids) and the time scale of the dynamical events of interest are longer than what molecular dynamics (MD) simulations can reach, typically a few nanoseconds. These methods are based on the hypothesis that the vibrational normal modes exhibiting the lowest frequencies (also named soft modes) describe the largest movements in a protein and are the ones functionally relevant.

Several tools based on NMA have been developed [1-16] and successfully applied to predict the collective, large amplitude motions of several macromolecules of different sizes, e.g. the F(1)-APTase[17], RNA polymerases[18] or bigger systems such as virus capsids[19]. Lately, web tools have appeared making this technique accessible to a larger number of users. The **elNémo**[20], web interface to the Elastic Network Model, offers normal modes calculations and a fairly large number of analyses for each calculated mode; degree of collectivity, animation (PDB downloadable files or animated GIF images) for each mode using three different views for the protein, comparison between experimental and predicted B-factors, maximum distance fluctuation between all pairs of Cα atoms and normalized mean squared atomic displacements. If two structures are uploaded, the cumulative overlap between the modes and the conformational difference is calculated. Delarue et al. [21] have developed another application based on the Elastic Network Model. The application offers calculations of normal modes on all atoms (the users can also choose to use only Cα) and provides an animation for each calculated mode (PDBmovies) that can be visualized with e.g. PyMol. The same group has developed a server performing normal modes calculations using a more general molecular mechanics force field, Gromacs, and which also provides animation of the vibrations corresponding to each calculated mode. The use of such a force field increases the computational cost of the computation and the system size is therefore limited to 5000 atoms. The **NMA movie generator**, available from the web pages of the database of macromolecular movements (MolMovDB[22]), calculates the five lowest frequency normal modes for a PDB structure file which can be either uploaded to the server or chosen by its PDB or SCOP identifiers. Animated GIF images of the vibrations are generated and compared with the pre-calculated flexibility regions based on supplied B-factors or multiple structural alignments for the corresponding fold family for one-domain fold proteins.

The Molecular Vibrations Evaluation Server (**MoVies**[23]) provides vibrational study of proteins and nucleic acids, using modified AMBER force field[24] and a self-consistent harmonic approximation method. Starting from a structure file in the PDB format, the application performs normal modes calculations and several analyses, and on completion the results are sent to the user by email. Of special interest is the evaluation of hydrogen bond disruption probability.

The **ProMode database **[25] is a database of normal mode analysis of proteins. Results of normal mode analysis for a large number of proteins are made accessible via a web interface. For each mode, an animation and the axes of the movement (as calculated by DynDom[26]) can be viewed using the Chime plugin. Fluctuations of atom positions and torsion angles, correlation between Cα atom displacements are plotted for each mode; the averages of these values over all modes are also stored in the database. Dynamical domains for each mode, characterized using DynDom, are given. Although NMA results for a large number of proteins can be very quickly retrieved from ProMode, not all proteins available in the Protein Data Bank are present and users cannot submit their own structure file.

We developed a web application for calculation of normal mode analysis which offers fast calculation of the 200 lowest frequency modes and different types of analyses: deformation energy, animation of the vibration, atomic squared displacements and vector field analysis. Results of each analysis can be visualized using only a web browser, without any additional plug-in or program. Alternatively, the users can download raw data and visualize them using their favorite software. We have carefully designed our web application into independent modules so that the users can perform only the analyses they are interested in, and in this way avoid spending time waiting for results of analysis irrelevant to their particular question. The modular structure will, in the future, allow us to easily add new functionality. The core of the application is written in the Python programming language, using the Molecular Modeling ToolKit [27] (MMTK). It contains an implementation of the approximate normal analysis method developed by Hinsen[10] which calculates low-frequency domain motions at negligible computational cost. Zope[28] is used for the web interface, which communicates with the core through an application server. Details of the implementation are given below, followed by an example calculation on a large transmembrane protein.

## Implementation

### 1. Web-interface

The first step for the user is to upload a pdb file containing the structure. Pressing the submit button starts the normal mode calculation, which runs to completion without doing any further analysis. No limit is set for the system size (i.e. number of residues). When the calculation is finished, the user is directed to a page which displays the result of the energy deformation analysis. Low average deformation energy indicates a mode with large rigid regions, i.e. a mode with a large degree of collectivity, which has a good chance of describing domain motions. This page is meant to help users judge for which mode(s), if any, the analysis will be significant in terms of large collective movements. They can then decide to perform further analysis of the calculated modes and are given the possibility to choose among three different analyses (see description below). Results of each analysis are stored and can at any time be viewed either in a separate window, or downloaded as a ZIP archive together with results of all other analyses performed up to that moment.

**Normalized squared atomic displacements **can be retrieved in two different formats. Users can download text files containing two columns, the first one corresponding to the amino acid numbers of the sequence in the structure file (PDB) submitted and the second one containing the normalized displacement corresponding to each amino acid. Alternatively, the user can retrieve PDF plots representing the variation of normalized atomic displacements vs. amino acid number. These plots are generated using the R programming language[29] and RPy [30], a Python interface to R. Thus, we provide the users with the possibility to see the results directly from their web browser without any additional plugins or program, but we also, for users who want to have more flexibility, provide the raw data.

**Mode animations **are provided for the six first significant modes (i.e. modes 7 to 12, see Methods section), as animated gif images or as DCD trajectory files. The DCD file format is a binary format for trajectories from MD simulations that is common to the CHARMm[1], XPlor[31] and NAMD[32] programs. DCD files can be read by VMD[33]. Unlike with animated gifs, visualizing DCD files with VMD allows the users to manipulate the protein themselves (rotate, zoom, highlight specific regions, etc..) which might offer a better insight in the calculated domain movements. On the other hand, this requires that the user has VMD installed on his computer and is sufficiently used to it. Therefore, we have decided to offer the possibility to choose the orientation of the protein before the animated gif images are generated. Rasmol[34,35] is used to generate image files of the different conformations along the mode vector (see Methods section). The images are then concatenated to produce an animation (animated GIF file) using Image Magick [36]. The resulting animation is a sequence of five conformations, with a delay of 1/25 second between them.

**Vector field representations **help characterize the domain displacements with vectors representing the direction and the relative displacements of the different regions of the protein. Using VMD, the web application generates a picture of the protein and the vectors for modes 7 to 12. Using the same setup as for the mode animations, the user can choose the orientation of his system. Additionally, VMD 'state' files are generated and available for download, allowing a more interactive inspection of the vector fields.

### 2. Application server

**Figure 1:**
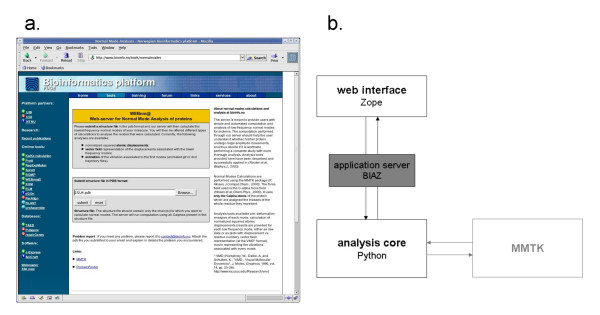
**WEBnm@ architecture. a. **The main page of WEBnm@ is a form where users can input a structure file in the PDB format. **b. **The server consists of two parts, the graphical web interface and the core of the program, written in Python, which performs the actual computation. The two parts communicate via a web application server, BIAZ.

The web interface of WEBnm@ is written using the DTML language of the Zope[28] webserver. The analysis core, written in Python, runs under the BIAZ application server[37]. BIAZ is connected to Zope using a pipe (see Figure 1). The purpose of the BIAZ application server is to simplify the development of web interfaces for computationally demanding applications; it has been developed and is used to run the computational services of the Norwegian Bioinformatics Platform . BIAZ itself is written in Common Lisp(CL), and applications in CL or Python are currently supported. The application server fetches the results after completion of the computation and sends them to the web interface (Zope). The division between core application and web interface also makes the code more readable, and thus maintainable. The core application code becomes thereby usable in other contexts as well.

## Results: example calculation on SERCA1 Ca-ATPase

The calcium ATPase from the sarcoplasmic reticulum, is constituted of 3 cytoplasmic domains, named Actuator (A, amino acids 1 to 40 (NTer) and 124 to 243), Nucleotidic (N, 360 to 604) and Phosphorylation (P, 330 to 359 and 605 to 737), and 10 transmembrane helices hosting the calcium binding sites. It is known that the cytoplasmic domains undergo large amplitude movements during the active transport of calcium ions. We recently reported a NMA study of the E1Ca form of the Ca-ATPase, starting from its x-ray structure (PDB ref 1EUL) [38]. Using MMTK, we could show that the N and A domains undergo the largest amplitude movements, as revealed by the lowest frequency modes. We highlighted a large amplitude movement of the transmembrane helices, which "twist-opens" the lumenal side of the protein.

**Figure 2:**
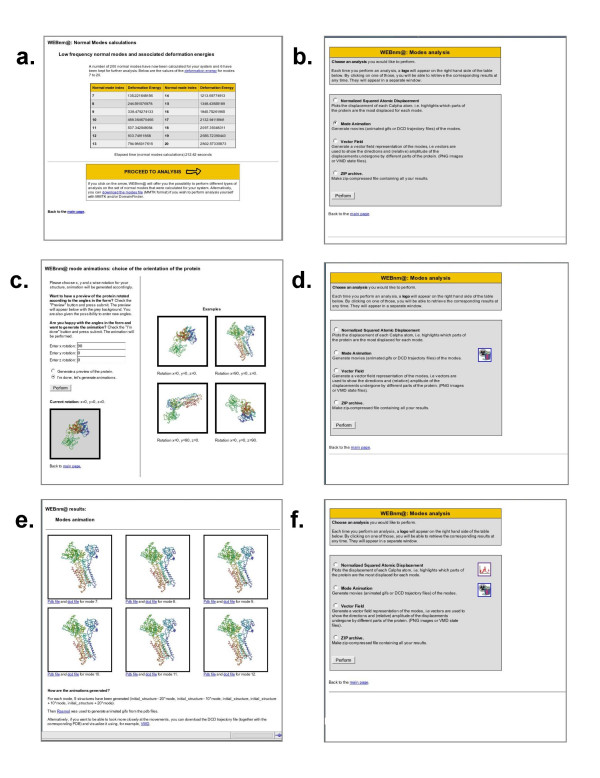
**Snapshots of an example calculation of the SERCA1 Ca-ATPase. a. **Presentation of the 10 lowest frequency modes with their average deformation energy. **b. **Page presenting the available analyses. **c. **Page where user can choose the orientation of the system for the animations. **d. **Apparition of an icon on the page presenting the list of analyses. **e. **Page displaying animated gif image of the first 6 modes (7 to 12). **f. **List of analyses page after that both mode animations and atomic displacements have been calculated, two icons are present.

In what follows, we explain how to use WEBnm@ to perform the same type of analysis (we use here the PDB ID 1SU4, instead of 1EUL) and especially how to interpret the results given by our application. We show that we obtain the same results with WEBnm@ as we obtained using a non automated procedure [38]. After the uploading of the structure file (PDB format) on the main page (Figure 1), normal modes are calculated. The server is directed to an html page with a table containing deformation energies for modes 7 through 20. The deformation energy of a mode is a measure of the collectivity of the movements associated to this mode. The lower the deformation energy, the higher the degree of collectivity. A high degree of collectivity means that large regions of the protein, possibly domains, are displaced. Although the deformation energies have no quantitative physical meaning (and therefore no unit), values obtained on different proteins can be compared. In our example (Cf. Figure 2a), the value of the deformation energy for the first mode is extremely low (135.2). In comparison, the deformation energy of the first mode for lysozyme is 2378.5 (pdb id: 153l), 795.0 for the MscL (pdb id: 1msl) and 5881.7 for trypsin (pdb id: 1anb), which is not known to undergo large amplitude domains movements.

The user can then choose to proceed to further analyis (Cf. Figure 2b), for example generate an animation for each of the 6 first modes (7 through 12). The next page (Figure 2c) offers the users the possibility to orient the system properly to ensure the best view of the movements by choosing a rotation angle over the x, y and z axes. A preview will be generated for each chosen set of angles. Once the user has decided upon a set of angles, he can check the 'I'm done' radio button, and then press the 'Perform' button and animations will be generated. The user is then brought back to the 'Analysis' page (Figure 2d) where a logo has now appeared next to 'Mode Animation'. By clicking on this icon, a new window containing the animated images (gif format) will be opened (Figure 2e). This goes for all additional analyses. A click on an icon opens a new window with the results of the corresponding analysis. At any moment, one can download the analyses performed up to that point as a ZIP archive that contains all result files.

**Figure 3:**
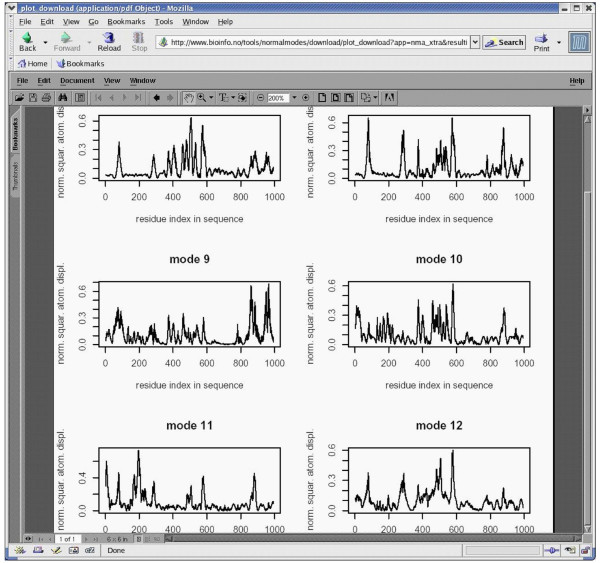
**Normalized atomic displacements plots. **Plots for modes 7 to 12 are generated on the same page and converted to a PDF file.

Figure 3 displays the plot obtained by calculating the normalized atomic squared displacements. For example, one can see that the displacements associated with modes 7 (top left plot) concern mostly the domain N (aminoacids number 360 to 604) and the L_1–2 _(aa 78 to 89), L_7–8_(aa 852 to 896) and L_9–10_(aa 949 to 965) loops.

## Conclusion

WEBnm@ allows efficient calculation of normal modes for proteins and is available to everyone from . Calculation of the modes for the Ca-ATPase, which contains 994 residues, takes about 4 minutes. Our web application has several other advantages; a user can choose which analyses to perform so that no time is wasted on analysis he/she is not interested in. Result pages for each analysis are independent and open in separate windows. All results are presented on the web pages, no additional programs or plugins are needed for visualization. However, results are also provided in other formats (x, y format for normalized squared atomic displacements, PDB for structure and DCD for trajectories) in case users want to use their favorite program to visualize and analyze their results. This allows anyone to calculate normal modes for relatively large systems, without having the required resources (i.e. memory) to do it in-house. At any time, result files of the calculation performed up to that moment can be downloaded in a ZIP file. Although WEBnm@ is not the first tool of his kind, it is probably the fastest and provides functionalities that are not found elsewhere.

The architecture of WEBnm@ is totally modular. It is meant to welcome an increasing number of functionalities (structure comparison between different conformations of a protein, domain determination, etc...). Decision on future developments will also be based on users' requests.

## Methods

### Normal modes calculations

A normal mode analysis (NMA) consists of the diagonalization of the matrix of the second derivatives of the energy with respect to the displacements of the atoms, in mass-weighted coordinates (Hessian matrix). The eigenvectors of the Hessian matrix are the normal modes, and its eigenvalues are the squares of the associated frequencies. We use the approximate normal modes calculation method developed by Hinsen [10] and implemented in the MMTK package[27]. This method represents the low-frequency domain motions very well at negligible computational cost. The force field used is slightly different from the one used in the original publication and has been described in reference [13]. It uses only the Cα atoms of the protein, which are assigned the masses of the whole residues they represent.

Briefly, the functional form of the force field is



V(r) is the harmonic pair potential describing the interaction between the Cα atoms:



where  is the pair distance vector (R_i _- R_j_) in the input configuration and k is the pair force constant:



Two hundred modes are calculated for proteins containing less than 1200 residues. For proteins containing more than 1200 residues, N/6 modes are calculated (N being the number of residues). The first six modes (zero-frequency modes) correspond to global rotation and translation of the system and are ignored in the analyses. Thus, the lowest frequency mode of interest is mode 7. Deformation energy and normalized atomic displacements analyses are performed for modes 7 through 20 while mode animations and vector fields are calculated for modes 7 through 12.

### Deformation energy

As in DomainFinder[10,11], a deformation energy is calculated for each atom. Deformation energy depends on the changes in the distance between the atom in question and each of its close neighbors. Low deformation energies indicate relatively rigid regions, whereas high deformation energies indicate flexible regions. The application returns the average deformation energy for each mode. Low average deformation energy indicates a mode with large rigid regions, which has a good chance of describing domain motions.

### Normalized squared atomic displacements

Normalized squared atomic displacements (D_i_) for each amino acid (resid) or Cα atom (i = 1 to n) are calculated as follows:



where d_i _is the component of the eigenvector corresponding to the *i*^*th *^residue.

### Normal mode animations

Subsequent structures of a given animation are generated by applying eigenvectors of the corresponding mode to the Cα coordinates of the structure submitted to the server. Two structures of the protein are generated in each direction (i.e. +a*mode, +2*a*mode, -a*mode, -2*a*mode). The 'a' factor is arbitrary; we choose to set it equal to 10 as a default value since this gives the best visual insight on the movements.

### Vector fields

A vector field representation is calculated as described by Thomas et al. [39]. The vector field is calculated over cubic regions with an edge length of 3 Å, containing on average 1.3 Cα atoms. The vector field defined on a regular lattice at the center of each cube is the mass-weighted average of the displacements of the atoms in the cube.

## Authors' contributions

SMH designed the modular architecture, developed the graphical presentation of results for mode animations and vector field representations, and served as the main driving force in building the latest version of WEBnm@. GS is the developer of the BIAZ application server; several features of BIAZ were developed especially for WEBnm@. NR wrote the core analysis code, designed and supervised the project, and edited the manuscript. This work is a truly collaborative effort of all three authors. All authors read and approved the final manuscript.
